# Physiologically based pharmacokinetic modeling of apixaban to predict exposure in populations with hepatic and renal impairment and elderly populations

**DOI:** 10.1007/s00228-023-03602-4

**Published:** 2023-12-15

**Authors:** Yichao Xu, Lei Zhang, Xiaofan Dou, Yongze Dong, Xiangchai Guo

**Affiliations:** 1 Center for Plastic & Reconstructive Surgery, Department of Orthopedics, Zhejiang Provincial People’s Hospital (Affiliated People’s Hospital), Hangzhou Medical College, Hangzhou, Zhejiang China; 2 Center of Clinical Pharmacology, the Second Affiliated Hospital of Zhejiang University, School of Medicine, Hangzhou, Zhejiang China; 3 Department of Pharmacy, the Second Affiliated Hospital of Zhejiang University, School of Medicine, Hangzhou, Zhejiang China

**Keywords:** Apixaban, Elderly population, Hepatic impairment, Physiologically based pharmacokinetics, Renal impairment

## Abstract

**Background:**

Apixaban is a factor Xa inhibitor with a limited therapeutic index that belongs to the family of oral direct anticoagulants. The pharmacokinetic (PK) behavior of apixaban may be altered in elderly populations and populations with renal or hepatic impairment, necessitating dosage adjustments.

**Methods:**

This study was conducted to examine how the physiologically based pharmacokinetic (PBPK) model describes the PKs of apixaban in adult and elderly populations and to determine the PKs of apixaban in elderly populations with renal and hepatic impairment. After PBPK models were constructed using the reported physicochemical properties of apixaban and clinical data, they were validated using data from clinical studies involving various dose ranges. Comparing predicted and observed blood concentration data and PK parameters was utilized to evaluate the model’s fit performance.

**Results:**

Doses should be reduced to approximately 70% of the healthy adult population for the healthy elderly population to achieve the same PK exposure; approximately 88%, 71%, and 89% of that for the elderly populations with mild, moderate, and severe renal impairment, respectively; and approximately 96%, 81%, and 58% of that for the Child Pugh-A, Child Pugh-B, and Child Pugh-C hepatic impairment elderly populations, respectively to achieve the same PK exposure.

**Conclusion:**

The findings indicate that the renal and hepatic function might be considered for apixaban therapy in Chinese elderly patients and the PBPK model can be used to optimize dosage regimens for specific populations.

**Supplementary Information:**

The online version contains supplementary material available at 10.1007/s00228-023-03602-4.

## Introduction

Direct oral anticoagulants (DOACs) are now recognized as the first-line treatment for preventing recurrent venous thromboembolism (VTE) and recurrent thromboembolic events in patients with atrial fibrillation [1, 2]. Apixaban is a narrow therapeutic index oral direct factor Xa inhibitor that belongs to the DOAC family. Apixaban 2.5 mg or 5 mg twice daily has been approved for preventing and treating VTE. Several clinical pharmacokinetic (PK) studies suggest that dose modification may be necessary for special populations, such as those with renal or hepatic impairment and the elderly, even though this universal dosing regimen may perform well on average [3–5].

Not only are elderly populations susceptible to atrial fibrillation (AF) in general, but also to specific forms of AF [6, 7]. Due to aging and complex underlying diseases treated by multidrug therapy, the pathophysiological mechanisms and pharmacokinetics of elderly populations are complex, and they face a substantially increased risk of bleeding with thrombosis treatment [8, 9]. Clinicians are increasingly concerned with balancing the benefits of anticoagulation and the increased risk of bleeding [10, 11]. Few randomized controlled studies have evaluated the risk of thrombosis and bleeding; the outcomes of anticoagulation therapy in elderly patients with AF at various ages, disease stages, and degrees of vulnerability; or the application of various anticoagulant medications [3]. It is essential to reduce the safety risk and maximize the efficacy-to-safety ratio of apixaban therapy in geriatric patients. To maintain the same efficacy and safety profile in elderly adults as in non-geriatric adults, it is necessary to predict the PK profile in virtual elderly populations.

A physiologically based pharmacokinetic (PBPK) model considers the physiological and biochemical properties of organisms and the physicochemical, anatomical, and thermodynamic properties of a drug [12–14]. This model simulates drug distribution, transportation, and metabolism in various body regions by treating human tissues and organs as independent compartments linked by blood circulation. To predict the PK and efficacy of drugs in humans, the PBPK model combines the physical and chemical properties of drugs, the parameters of the human physiological system, and the mechanical PK data [15–18]. Consequently, it can process medical dynamics data based on the principle of material equilibrium [19]. In addition, PBPK is frequently used to characterize PK alterations in the body under various complex clinical conditions and, according to previous research, is an effective method for examining the distribution and metabolism of drugs in the human body [20].

In this study, we followed the methods of Shen et al. [21]. A PBPK model was developed and validated for extrapolation to the healthy elderly Chinese population and the elderly Chinese population with hepatic and renal impairment to serve as a guide for devising individualized medication regimens for these populations.

## Methods

### Modeling platform and data collection

The population-based PBPK simulator PK-Sim^®^ software version 11.1 (Open Systems Pharmacology Suite) was utilized to construct whole-body PBPK models of apixaban in healthy adults, healthy elderly, and elderly populations with hepatic and renal impairment. The mean concentration versus time data points were extracted using version 4.2 of the open-source WebPlotDigitizer^®^ software. The non-compartmental model analysis software Phoenix WinNonlin^®^ software version 8.3.5.340 (Pharsight, Mountain View, CA, USA) was used to calculate PK parameters.

### Generic workflow for model development

**Fig. 1:**
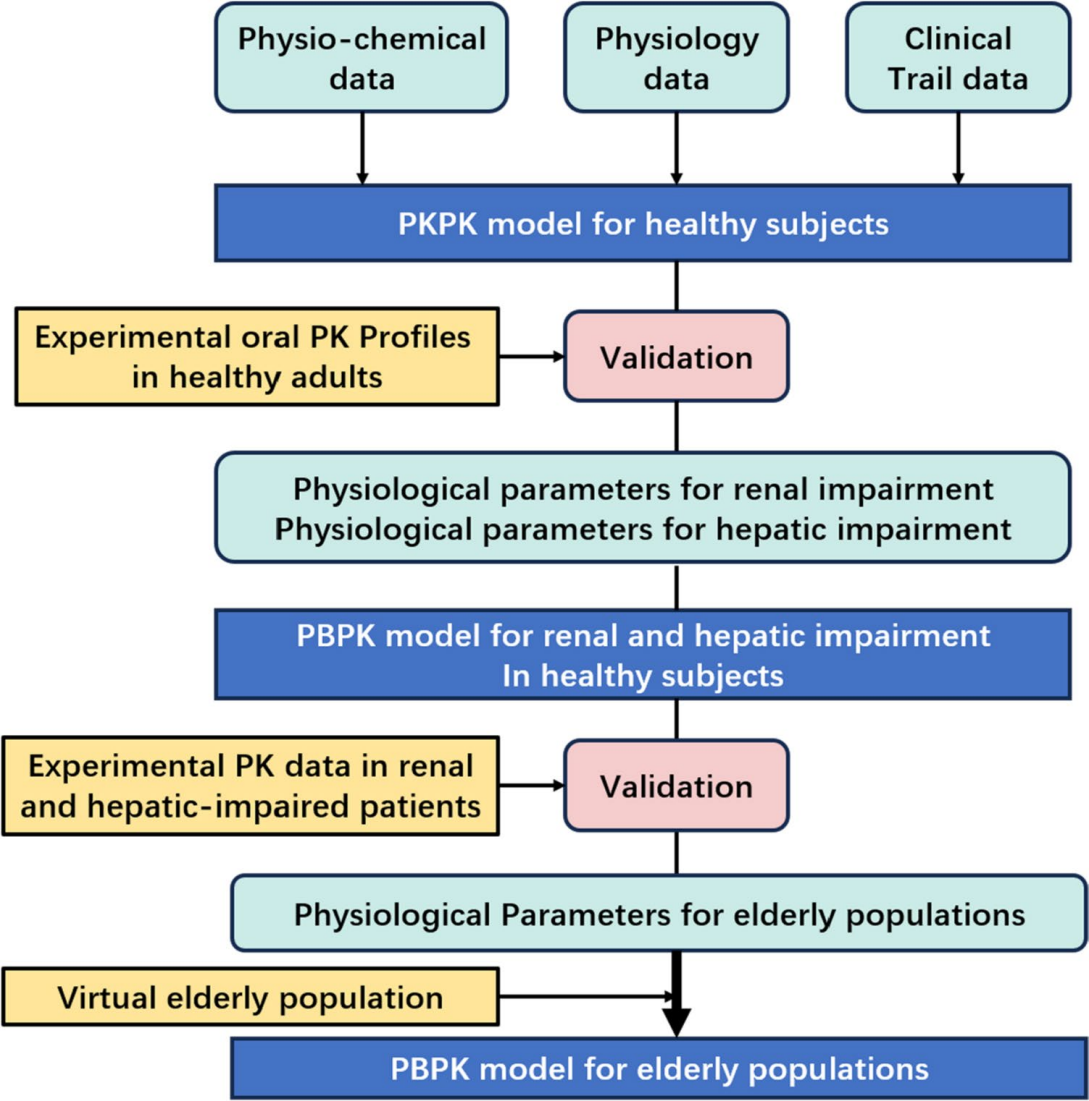
Generic workflow for model development

Figure 1 depicts the generic workflow for scaling the pharmacokinetics of apixaban from healthy adults to populations with hepatic and renal impairment and from healthy adults to geriatric adults using PBPK modeling.

### Adult PBPK model development

This study utilized a combined “bottom-up” and “middle-out” strategy to facilitate model development. The ADME mechanism of the drug apixaban was developed by accumulating information on its physicochemical properties, conducting in vitro experiments, and extending the experiments to humans with ex vivo correlated factors and scalars [22]. The final model was created using the software PK-Sim’s 18 compartments, each of which could be further subdivided into sub-compartments [23]. Compound parameters were obtained from the DrugBank website, US Food and Drug Administration (FDA) medication guidelines and literature, and European Medicines Agency (EMA) medication guidelines and literature [3, 4, 24–29]. Physicochemical properties, intestinal permeability, enzymatic kinetics, and glomerular filtration fraction (f_GFR_) parameters were analyzed. In constructing the initial model, system-specific parameters derived from the PK-Sim built-in database (i.e., physiological and anatomical parameters of the virtual population) were fixed using Monte Carlo simulations to suit oral tablet clinical data for 2.5 mg.

The model was validated using data from other single- and multiple-dosing regimens [24, 30]. Table 1 summarizes the physicochemical, biopharmaceutical, and PK parameters of apixaban.

**Table 1:** Summary of input compound parameters of apixaban PBPK model

**Parameter**	**Apixaban**	**Source**
Physiochemical properties		
log P	2.22	DrugBank
f_u_ (plasma, albumin)	0.93	[27]
MW (g/mol)	459	DrugBank
pKa (acid)	13.07	DrugBank
Absorption	9E-07	[28]
Specific intestinal		
permeability (cm/min)		
Distribution		
Partition coefficients	PK-Sim Standard	[29]
Cellular permeabilities	PK-Sim Standard	[29]
Metabolism and elimination		
CL_H_ (L/h)	2.4	[3]
f_GFR_	0.18	Calculation

*CL*_*H*_ hepatic clearance, *f*_*GFR*_ glomerular filtration rate fraction, *f*_*u*_ fraction unbound, *log P* lipophilicity, *MW* molecular weight, *pKa* acid dissociation constant, *PBPK* physiologically based pharmacokinetic

#### PBPK modeling in healthy adults

Based on the software’s calculated mean values, a virtual European adult was constructed to represent the population’s average adult. According to the mean population values, the individual’s age, weight, height, and body mass index (BMI) were 30.00 years, 82.00 kg, 180.00 cm, and 25.31 kg/m^2^, respectively. Using the “population” module of the software, a virtual population of six males aged 25 to 35 years was created to characterize the PK behavior of apixaban in the population. Based on the dosage regimen, all virtual populations were generated, and the population prediction means and 5th–95th concentration range were obtained. The characteristics of the clinical data of European healthy adults used in model development are shown in Table S1. Visual inspection was used to evaluate the predictive performance of the model by comparing the predicted and observed values.

Using the average folded error (AFE) method, the predicted concentrations were compared to the measured concentrations, and the maximum concentration (C_max_) and area under the curve from zero to infinity (AUC_0-∞_) were used to evaluate the model fit [31].

#### Scaling in renal-impairment populations

Based on the glomerular filtration fraction (f_GFR_) and creatinine clearance rate (CL_cr_), renal clearance in populations with renal impairment was predicted. Chang et al.’s reported real-world PK study was used to validate the model [32], and the characteristics of the clinical data of European patients with renal impairment used in model development are shown in Table S2 using the formula

1 $${f}_{GFR}=\frac{{CL}_{R}}{{f}_{u}\times GFR},$$

2 $${CL}_{R,i}={CL}_{R,j}\times \frac{{CL}_{cr,i}}{{CL}_{cr,j}},$$

where f_GFR_ represents the glomerular filtration fraction, CL_R_ represents observed renal clearance, fu represents the fraction unbound, and GFR represents the glomerular filtration rate.

#### Scaling in hepatic impairment populations

In populations with hepatic impairment, the Child–Pugh classification is the most prevalent method for classifying hepatic function. Patients are classified into Child-Pugh-A (CP-A), Child-Pugh-B (CP-B), and Child-Pugh-C (CP-C) groups based on the severity of hepatic impairment [33]. Table 2 provides information regarding parameters, and the characteristics of the clinical data of European patients with hepatic impairment used in model development are shown in Table S3. Frost et al.’s report on a real-world PK investigation served as the basis for model validation [34].

**Table 2:** Changes in PBPK parameters that are altered in hepatic impaired individuals versus healthy individuals

**Parameter**	**Healthy**	**CP-A**	**CP-B**	**CP-C**
Blood flow rate (L/min)				
Hepatic	0.44	0.45	0.79	0.15
Renal	1.35	0.94	0.69	0.51
Other organs (fractions of healthy)	1	1.75	2.25	2.75
Liver volume (L)	2.44	1.32	1.05	0.53
Hematocrit	0.47	0.39	0.37	0.35
Ontogeny factor (albumin)	1	0.81	0.68	0.5
Ontogeny factor (α1-acid glycoprotein)	1	0.6	0.56	0.3

### Chinese elderly population PBPK model development

The scaling of Chinese elderly populations was accomplished in two stages. In the first stage, PK-Sim was used to autonomously scale the parameters of anthropometric, anatomical, and physiological changes based on the final PBPK model for adults while keeping drug-specific parameters constant. Based on the prevalence of hepatic and renal dysfunction in this population, the second stage involved simulating apixaban exposure in subpopulations of the Chinese elderly population with hepatic and renal impairment separately.

### Analysis of model predictability

The prediction accuracy was evaluated graphically by comparing the in vivo observed concentration-time profiles with the simulated profiles. Non-compartmental analysis was utilized to derive the predicted PK parameters (AUC and C_max_) from simulated plasma concentration-time profiles. The AUC values were calculated using the linear trapezoidal rule and extrapolation to infinity. The C_max_ values were derived directly from the concentration-time profiles of plasma.

### Dosage optimization

Based on simulated target exposures (i.e., AUC and C_max_) in adults, dosing regimens for apixaban in various populations were evaluated. In particular, each individual’s calculation of AUC values was approximated by creating and grouping various categories of special virtual populations and selecting the optimal clinical dose for dose normalization. A comparison was made between the differences in AUC between each group and the adult group, and the data were annotated. Based on dose-normalization results for adults and special populations, the concentrations were adjusted to attain exposure levels comparable to those of healthy adults. For each type of special population, dosing recommendations were made.

## Results

### Prediction of PK profiles of apixaban in healthy adult subjects

**Fig. 2:**
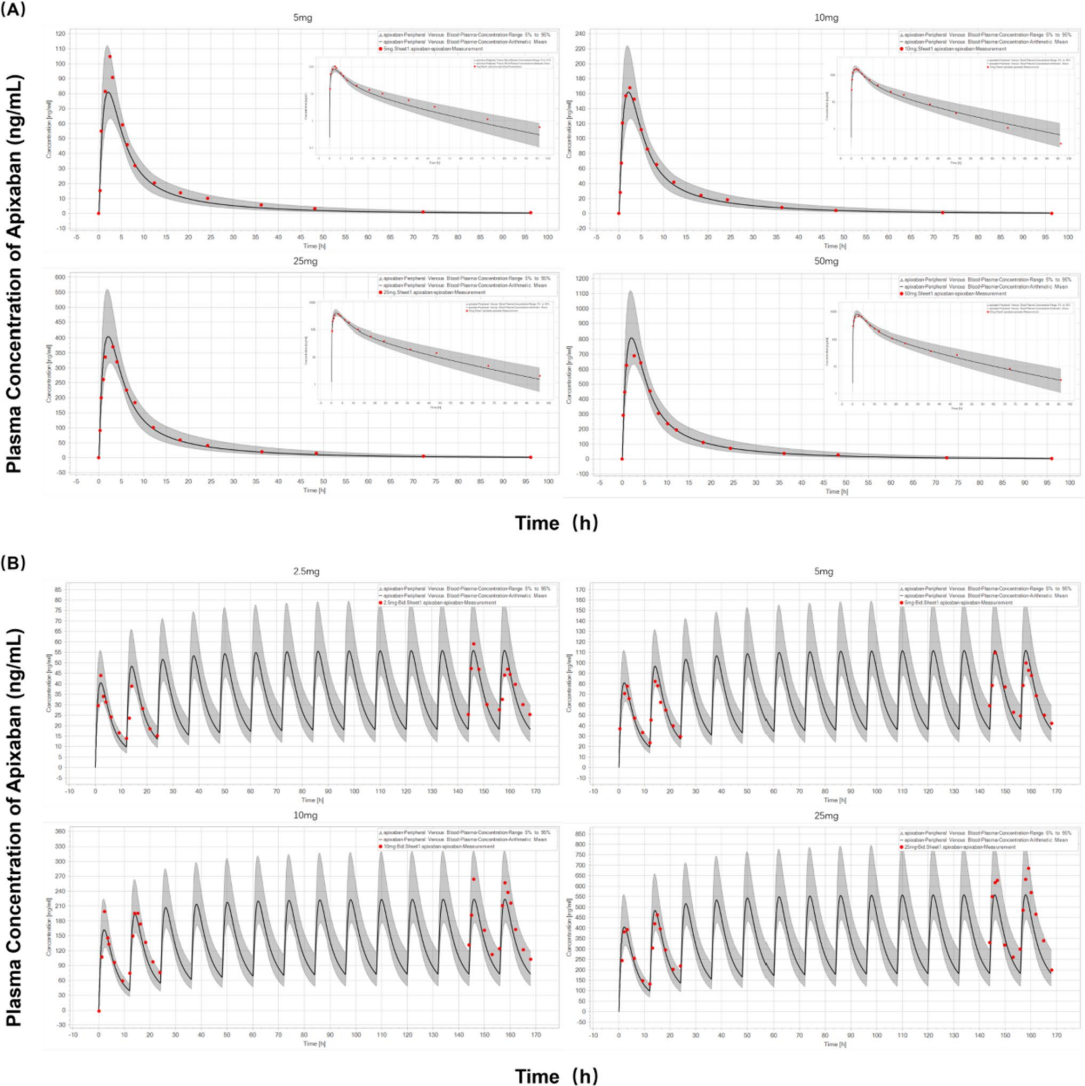
Prediction of the pharmacokinetic profiles for apixaban at a series of doses in healthy adults using physiologically based pharmacokinetic modelling. Simulation (mean predictions in black lines and 5th–95th percentiles of prediction in grey shade) of pharmacokinetic profiles for a single oral dose of 5, 10, 25, and 50 mg of apixaban (log scale was on the right top in each dose panel) (**A**) and multiple doses of 2.5, 5, 10, and 25 mg of apixaban (**B**). Simulations were compared with the corresponding observed clinical data of 6 healthy subjects (red dot), which were collected from the single- and multiple-dosing PK study of apixaban by Forst et al.

Firstly, the PBPK model was used to simulate the concentration-time profiles of apixaban in healthy adults after single and multiple administrations of varying dosages. For single-dosage simulation, 5 mg, 10 mg, 25 mg, and 50 mg of apixaban were administered to healthy participants in each dose group. For the multiple-dose simulation, healthy subjects were administered twice daily doses of 2.5, 5, 10, or 25 mg of apixaban for 7 days. As depicted in Fig. 2, the model precisely characterized the observed PK profile across all investigated dosage regimens. Moreover, accurately predicted are the terminal elimination after a single dose and the accumulation after multiple doses. These results indicate that a plausible absorption and elimination mechanism for apixaban can be assumed by the PBPK model.

### Prediction of PK profiles of apixaban in the renal-impairment population

**Fig. 3:**
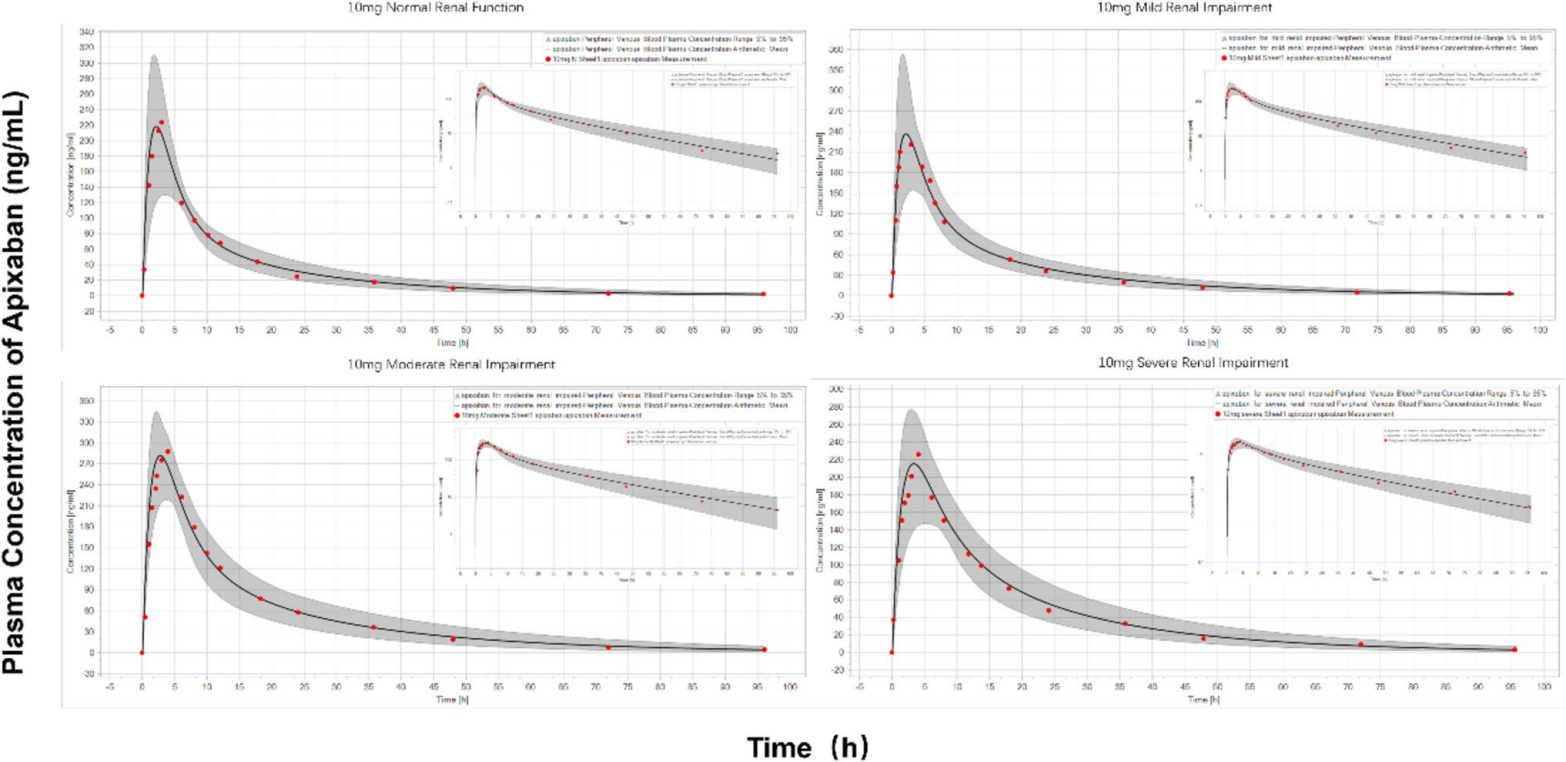
Prediction of the pharmacokinetic profiles of apixaban in healthy adults and patients with different renal impairments using physiologically based pharmacokinetic modeling. Simulations were compared with the corresponding observed clinical data (red dot), which were collected from the PK study of apixaban in patients with differing degrees of renal impairment by Chang et al. (8 patients with normal renal function, 10 patients with mild renal impairment, 7 patients with moderate renal impairment, 7 patients with severe renal impairment)

Based on data from a PK study of apixaban in patients with differing degrees of renal impairment, the PK characteristics of apixaban in the renal-impaired population were investigated [32]. As depicted in Fig. 3, the results of the extrapolation model demonstrated that the predicted and observed values of plasma drug concentration-time profiles in populations with mild, moderate, and severe renal impairment suit well, with the majority of observations lying within the 5th–95th percentile. The effect of renal impairment on the fold changes of apixaban’s C_max_ and AUC_0-∞_ was predicted and found to be comparable to the observed values in the group of patients with renal impairment in Table 3. To obtain the same therapeutic effect, the doses for patients with mild, moderate, and severe renal impairment must be reduced to approximately 76%, 53%, and 75% of the doses for healthy adults.

**Table 3:** Fold changes of apixaban exposure in adults with renal impaired compared with normal function

**Renal impairment**	**Observed** **C**_**max**_ **ratio**	**Observed** **AUC ratio**	**Predicted** **C**_**max**_ **ratio**	**Predicted** **AUC ratio**
Normal (CLCR = 120 mL/min)	1.00	1.00	1.00	1.12
Mild (CLCR = 65 mL/min)	1.02	1.30	1.05	1.32
Moderate (CLCR = 40 mL/min)	1.29	1.77	1.28	1.87
Severe (CLCR = 15 mL/min)	0.94	1.27	0.98	1.33

### Prediction of PK profiles of apixaban in the hepatic impairment population

**Fig. 4:**
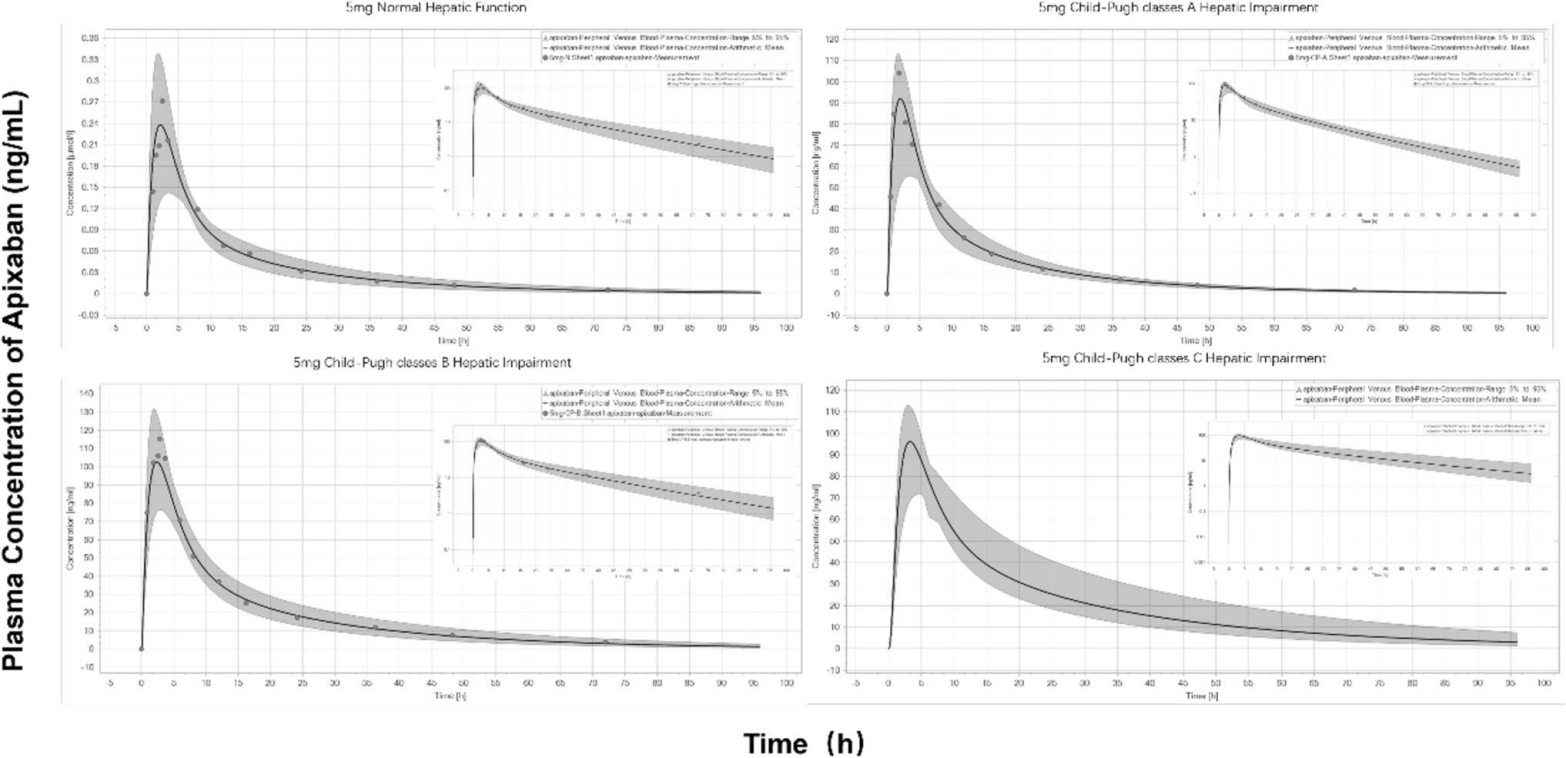
Prediction of the pharmacokinetic profiles of apixaban in healthy adults and patients with different hepatic impairments using physiologically based pharmacokinetic modeling. Simulations were compared with the corresponding observed clinical data (red dot) which were collected from the PK study of apixaban in patients with differing degrees of hepatic impairment by Forst et al. (16 patients with normal hepatic function, 8 patients with mild hepatic impairment, 8 patients with moderate hepatic impairment)

Using data from a PK study of apixaban in patients with differing degrees of hepatic impairment, the PK characteristics of apixaban in the hepatic-impaired population were investigated further [34]. As depicted in Fig. 4, the results of the extrapolation model indicated that the predicted and observed values of plasma drug concentration-time profiles in the CP-A and CP-B hepatic-impaired populations fit well, with most observations lying within the 5th–95th percentile. Based on this discovery, plasma drug concentration-time curve profiles in CP-C populations with hepatic impairment were predicted. The effect of hepatic impairment on the fold alterations of apixaban’s C_max_ and AUC_0-∞_ was predicted and comparable to the observed values in patients with hepatic impairment in Table 4. To obtain the same therapeutic effect, the doses for patients with CP-A, CP-B, and CP-C hepatic impairment must be reduced to approximately 96%, 88%, and 54% of the doses for healthy adults.

**Table 4:** Fold changes of apixaban exposure in adults with hepatic impaired compared with normal function

**Hepatic impairment**	**Observed** **C**_**max**_ **ratio**	**Observed** **AUC ratio**	**Predicted** **C**_**max**_ **ratio**	**Predicted** **AUC ratio**
Normal	1.00	1.00	0.91	1.05
Mild (CP-A)	0.85	1.03	0.76	1.04
Moderate (CP-B)	0.93	1.09	0.84	1.13
Severe (CP-C)	NA	NA	0.79	1.86

### Prediction of PK profiles of apixaban in the elderly Chinese population

**Fig. 5:**
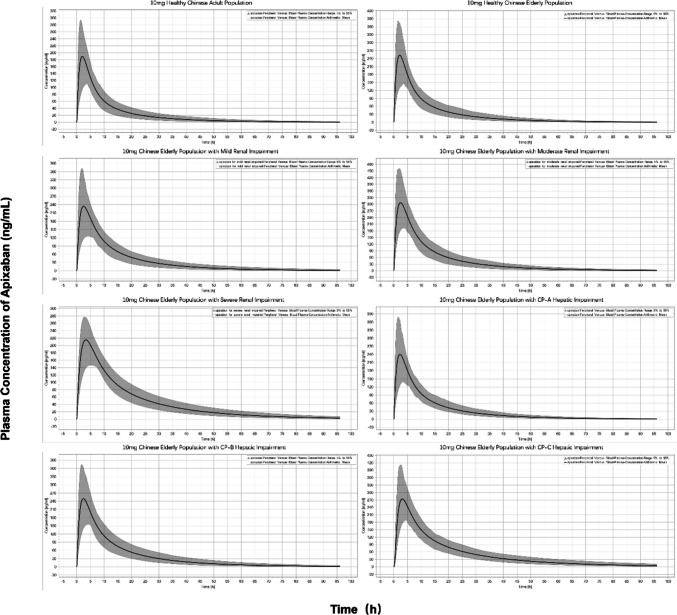
Comparison of oral 10 mg apixaban plasma drug concentration-time profiles in adult and elderly populations at different disease stages

Using the software’s built-in algorithm to scale the age-dependent parameters, a cohort of elderly individuals (50% women) aged 60 to 81 years was created. Exposure to 10 mg apixaban orally administered in vivo was predicted for the healthy geriatric Chinese population compared to the healthy adult Chinese population (50% females aged 20–40 years). As shown in Fig. 5, the AUC_0-∞_ and C_max_ of 10 mg of apixaban administered orally to healthy Chinese adult and elderly Chinese populations were 2062 and 2933 ng·h·mL^−1^ and 189.5 and 254 ng·mL^−1^, respectively. These results suggested that the in vivo dosage of apixaban administered orally to healthy Chinese elderly patients should be reduced to approximately 70% of the dose administered to healthy Chinese adults. The reaction to oral administration of 10 mg of apixaban was also predicted in elderly populations with hepatic and renal impairment. The results demonstrated that the AUC_0-∞_ of the elderly population with mild, moderate, and severe renal impairment was 3316, 4151, and 3285 ng·h·mL^−1^, respectively, and that the dosage for these populations should be reduced to approximately 88%, 71%, and 88%, respectively, of the dosage for the healthy elderly population to achieve the same therapeutic effect. Similarly, the AUC_0-∞_ of the elderly with CP-A, CP-B, and CP-C hepatic impairment was 3051, 3606, and 5059 ng·h·mL^−1^, and the dosage for these populations should be reduced to approximately 96%, 81%, and 58% of the dosage for the healthy population. Table 5 displays the predicted fold changes of AUC_0-∞_ and C_max_ of apixaban in Chinese elderly patients with renal/hepatic impairment.

**Table 5:** Fold changes of predicted candesartan exposure in Chinese elderly patients with renal or hepatic impaired compared with healthy subjects

	**Healthy subjects**	**Healthy elderly patients**	**Elderly patients with renal impairment**				**Elderly patients with hepatic impairment**	
			**Mild**	**Moderate**	**Severe**	CP-A	CP-B	CP-C
**C**_**max**_** ratio**	1.00	1.32	1.24	1.62	1.14	1.27	1.32	1.45
**AUC ratio**	1.00	1.42	1.61	2.01	1.59	1.48	1.75	2.45

## Discussion

The need for clinical anticoagulation treatment in populations has increased in recent years. Due to the limitations of conventional agents such as unfractionated heparin, low molecular weight heparin, and warfarin, DOACs such as apixaban have demonstrated promise in special populations, including those with renal or hepatic impairment and geriatric. The potential advantages of apixaban over conventional agents include its simplicity of administration, decreased frequency of drug monitoring, and reduced drug-food interactions. Due to a lack of clinical data for special populations, it must be extrapolated from the dosing regimen used in healthy adults to determine the dosage of apixaban that should be administered for off-label use.

Traditional compartmental modeling approaches have limited predictive ability because they do not account for all physiological, anatomical, and biochemical changes associated with drug exposure, nor all changes associated with drug absorption, distribution, metabolism, and excretion. In contrast, PBPK modeling utilizes existing drug disposition and physiology knowledge and enables extrapolation across various life stages. This study used PBPK-based scaling from healthy populations to populations with renal or hepatic impairment and from adults to children to predict apixaban PK profiles for elderly populations with or without renal or hepatic impairment. Utilizing age-specific physiology parameters, such as organ volume, blood flow, and hepatic and renal function, the model was then extrapolated to geriatric populations using adult-specific data.

Compared to traditional PK methods, PBPK models significantly impact the formulation of clinical medication regimens for special populations, primarily by predicting plasma concentrations of pharmaceuticals and providing an accurate method for assessing efficacy and risk [35, 36]. PBPK models have been used to predict exposure profiles in special populations in vivo due to their adaptability in data integration and excellent predictive potential [37]. We have experience with PBPK modeling and have developed accurate PBPK models for various innovative and generic medications [38, 39]. This was the first study to construct PBPK models for apixaban exposure in populations with hepatic and renal impairment and in Chinese elderly populations. The PBPK model specific for apixaban exposure in the population with hepatic and renal impairment was established and validated by relevant clinical studies by considering the differences in physiological characteristics between different disease stages and healthy populations. In contrast, the reactions to exposure in the elderly population were described based on age-scaling-related parameters. This model was extrapolated to older populations with impaired hepatic and renal function. The results of this model suggest that the PBPK model could be used to guide the clinical development of dosing regimens for apixaban in special populations.

In terms of substance PKs, both the liver and kidneys play a crucial role. The bioavailability of a drug is significantly influenced by the amount of drug absorbed and the liver’s first-pass metabolism. In contrast, the GFR is primarily responsible for eliminating the drug. Determining drug dosages for hepatic and renal insufficiency populations has been a significant clinical challenge [40]. Because apixaban is partially excreted through the kidneys (approximately 27% of absorbed apixaban), patients with renal impairment may experience increased systemic exposure [41], whose metabolism is mediated by the liver enzymes CYP3A4 and CYP3A5. Therefore, dosage adjustments must be made for these populations. In the current study, all in vivo parameters associated with renal and hepatic impairment were accounted for so that PBPK models for the renal- and hepatic-impaired populations could be extrapolated, and the dosage for these populations could be adjusted, with the adjustments supplemented by normalization of in vivo exposure in adults.

Due to the low availability of recruiting the elderly patients, clinical drug development has historically favored younger and middle-aged adults. Although those aged 65 and older receive the majority of drug prescriptions, they continue to be underrepresented in clinical trials [42]. Consequently, there is a lack of knowledge regarding the PK and PD responses of the elderly, rendering the safety and efficacy of pharmaceuticals in this population uncertain [43]. To contribute to its elucidation, this study extrapolated the reaction of healthy individuals to apixaban exposure to describe the reaction of healthy elderly and elderly populations with hepatic and renal impairment. The results indicate that elderly populations with hepatic and renal impairment should adjust their dosages on a similar scale to that of the healthy elderly population.

Although the PBPK model developed in this study has a stable structure and excellent predictive ability, several limitations must be considered when evaluating the results. One limitation is that all data used were extracted using software from published literature. Even though these data were not obtained directly from the researchers, the PK parameters calculated from these extracted data points were comparable to previously reported PK parameters, for which minor errors were inevitable but within acceptable limits. Another limitation is that as the only the immediate-release formulation of apixaban was considered when devising the PBPK model, the model only provides a generalized explanation of apixaban’s PKs at various oral dosages. Moreover, the PBPK we developed in this study was used to predict the dosage to achieve the same PK exposure rather than performing the same clinical efficacy. Unfortunately, there is no real-world PK study reported on the elderly Chinese patient population so far. In this case, we simulated PK exposure in the Chinese special patient population with the PBPK model to reflect the clinical efficacy for clinical use. Until now, what we had done was just a reference for the clinical use of apixaban in the Chinese patient population and we will continue to focus on this issue and verify our predictions in follow-up studies. Using the findings of this study, however, the model can be further evaluated and optimized in future studies.

## Conclusion

The PBPK model developed in this investigation contributes to a more accurate description of apixaban’s PKs in elderly populations with renal and hepatic impairment. By incorporating changes in pathophysiological factors into the model and accurately extrapolating the model to these populations, this study demonstrated a method for enhancing the predictive capacity of the drug-disease model, thereby making it a valuable resource for future clinical individualized drug administration and evaluation.

### Supplementary Information

Below is the link to the electronic supplementary material.

Supplementary file1 (DOCX 26 KB)

Supplementary file2 (ZIP 44536 KB)

## Data Availability

All data and materials included in this study are available upon request by contact with the corresponding author.
