# Supplementary material for: Physiologically based pharmacokinetic modeling of apixaban to predict exposure in populations with hepatic and renal impairment and elderly populations
Source: Eur J Clin Pharmacol. 2023 Dec 15;80(2):261–71. doi: 10.1007/s00228-023-03602-4 (PMC10847219; doi:10.1007/s00228-023-03602-4)
Supplement: Supplementary file 1 — Supplementary file1 (DOCX 26 KB) [file 228_2023_3602_MOESM1_ESM.docx]

**SUPPLEMENTARY MATERIALS**

**Physiologically based pharmacokinetic modeling of apixaban to predict exposure in populations with hepatic and renal impairment and elderly populations**

Yichao Xu, Lei Zhang, Xiaofan Dou, Yongze Dong and *Xiangchai Guo

* Corresponding Author: Xiangchai Guo

Center for Plastic & Reconstructive Surgery, Department of Orthopedics

Zhejiang Provincial People's Hospital, Hangzhou Medical College

Address: 158 Shangtang Road, Hangzhou, Zhejiang, 310009, China

Tel: +86 0571 87783759

Fox: +86 0571 87783969

Email: [zdhgxc@sina.com](mailto:zdhgxc@sina.com)

**Table S1.** Characteristics of the clinical data of adults used in model development.

| **References** | **Administration protocol** | **Sample Size (N)** | **Age**  **(Years)** | **Weight**  **(kg)** | **BMI**  **(kg/m^2^)** |
| --- | --- | --- | --- | --- | --- |
|  |  |  | Mean±SD | Mean±SD | Mean±SD |
| Frost 2013^1^ | PO 5 mg | 6 | 30±7 | 82.4±11.4 | 25.4±1.9 |
|  | PO 10 mg | 6 | 27±3 | 73.8±11.5 | 23.2±3.2 |
|  | PO 25 mg | 6 | 32±7 | 73.3±6.2 | 24.5±0.9 |
|  | PO 50 mg | 6 | 30±9 | 77.0±12.2 | 25.8±2.9 |
| Frost 2013^2^ | PO 2.5 mg BID | 6 | 27±7 | 78.3±13.9 | 24.4±2.1 |
|  | PO 5 mg BID | 6 | 31±6 | 72.3±10.4 | 23.2±3.2 |
|  | PO 10 mg BID | 6 | 30±9 | 70.5±9.6 | 23.7±3.1 |
|  | PO 25 mg BID | 6 | 27±8 | 82.3±5.8 | 25.5±1.2 |

**Table S2** Characteristics of the clinical data of the renal impairment population used in model development.

| **Reference** | **Disease status** | **Administration protocol** | **MDRD**  **eGFR (mL/min/1.73 m^2^)**  **Median [range]** | **Sample Size (N)** | **Age**  **(Years)**  **Mean±SD** | **Weight**  **(kg)**  **Mean±SD** | **BMI**  **(kg/m^2^)**  **Mean±SD** |
| --- | --- | --- | --- | --- | --- | --- | --- |
| Chang 2016^3^ | Healthy | PO 10mg | 80.7 [72.3-89.3] | 8 | 59±2 | 83.8±9.7 | 29.0±3.8 |
|  | Mild | PO 10mg | 49.3 [29.4-70.2] | 10 | 61±13 | 79.3±16.3 | 29.4±4.4 |
|  | Moderate | PO 10mg | 44.7 [18.1-74.0] | 7 | 68±11 | 74.5±14.7 | 26.6±3.7 |
|  | Severe | PO 10mg | 19.2 [13.8–24.2] | 7 | 65±7 | 84.1±23.9 | 29.6±7.5 |

**Table S3** Characteristics of the clinical data of the hepatic impairment population used in model development.

| **Reference** | **Disease status** | **Administration protocol** | **Sample Size (N)** | **Age (Years)**  **Mean±SD** | **Weight (kg)**  **Mean±SD** | **BMI (kg/m^2^)**  **Mean±SD** |
| --- | --- | --- | --- | --- | --- | --- |
| Frost 2021^4^ | Healthy | PO 5 mg | 16 | 48±8 | 77.6±10.2 | 27.4±3.3 |
|  | CP-A | PO 5 mg | 8 | 52±7 | 86.1±16.3 | 29.0±3.3 |
|  | CP-B | PO 5 mg | 8 | 50±5 | 83.8±19.7 | 28.4±4.5 |

Reference:

1. Frost C, Wang J, Nepal S, et al. Apixaban, an oral, direct factor Xa inhibitor: single dose safety, pharmacokinetics, pharmacodynamics and food effect in healthy subjects. Br J Clin Pharmacol. 2013;75(2): 476-487.

2. Frost C, Nepal S, Wang J, Schuster A, Byon W, Boyd RA, et al. Safety, pharmacokinetics and pharmacodynamics of multiple oral doses of apixaban, a factor Xa inhibitor, in healthy subjects. Br J Clin Pharmacol. 2013;76(5):776–86.

3. CHANG M, YU Z, SHENKER A, et al. Effect of renal impairment on the pharmacokinetics, pharmacodynamics, and safety of apixaban [J]. J Clin Pharmacol, 2016, 56(5): 637-645.

4. FROST C E, LY V, GARONZIK S M. Apixaban Pharmacokinetics and Pharmacodynamics in Subjects with Mild or Moderate Hepatic Impairment [J]. Drugs R D, 2021, 21(4): 375-384.
